# Characterizing the relationship between peak assistance torque and metabolic cost reduction during running with ankle exoskeletons

**DOI:** 10.1186/s12984-022-01023-5

**Published:** 2022-05-12

**Authors:** Delaney E. Miller, Guan Rong Tan, Emily M. Farina, Alison L. Sheets-Singer, Steven H. Collins

**Affiliations:** 1 Department of Mechanical Engineering, Stanford University, Stanford, CA USA; 2 Sports Research Laboratory, Nike Inc., Beaverton, OR USA

**Keywords:** Exoskeleton, Augmentation, Running, Human-in-the-loop optimization, Metabolic cost

## Abstract

**Background:**

Reducing the energy cost of running with exoskeletons could improve enjoyment, reduce fatigue, and encourage participation among novice and ageing runners. Previously, tethered ankle exoskeleton emulators with offboard motors were used to greatly reduce the energy cost of running with powered ankle plantarflexion assistance. Through a process known as “human-in-the-loop optimization”, the timing and magnitude of assistance torque was optimized to maximally reduce metabolic cost. However, to achieve the maximum net benefit in energy cost outside of the laboratory environment, it is also necessary to consider the tradeoff between the magnitude of device assistance and the metabolic penalty of carrying a heavier, more powerful exoskeleton.

**Methods:**

In this study, tethered ankle exoskeleton emulators were used to characterize the effect of peak assistance torque on metabolic cost during running. Three recreational runners participated in human-in-the-loop optimization at four fixed peak assistance torque levels to obtain their energetically optimal assistance timing parameters at each level.

**Results:**

We found that the relationship between metabolic rate and peak assistance torque was nearly linear but with diminishing returns at higher torque magnitudes, which is well-approximated by an asymptotic exponential function. At the highest assistance torque magnitude of 0.8 Nm/kg, participants’ net metabolic rate was 24.8 ± 2.3% (p = 4e–6) lower than running in the unpowered devices. Optimized timing of peak assistance torque was as late as allowed during stance (80% of stance) and optimized timing of torque removal was at toe-off (100% of stance); similar assistance timing was preferred across participants and torque magnitudes.

**Conclusions:**

These results allow exoskeleton designers to predict the energy cost savings for candidate devices with different assistance torque capabilities, thus informing the design of portable ankle exoskeletons that maximize net metabolic benefit.

**Supplementary Information:**

The online version contains supplementary material available at 10.1186/s12984-022-01023-5.

## Background

Increasing global participation in physical activity, specifically running, could reduce mortality rates and improve individuals’ physical and mental health [1–4]. However, the metabolic demands of running can make it difficult for novice and ageing runners to engage with the activity. Robotic assistance in the form of portable, wearable exoskeletons has the potential to increase accessibility to and interest in running by reducing the energy required to participate in the sport. Reducing energy cost would likely also reduce perceived exertion and could improve confidence and perceived competence, both of which are associated with increased enjoyment and frequency of exercise [5, 6]. Allowing runners to match the pace of fitter friends could also leverage the benefits of exercising with others [7–9].

Recently, various wearable technologies have been developed that successfully reduce the energy cost of running. While passive systems can lower energy cost by moderate amounts, powered exoskeleton devices have the potential to provide larger reductions in the metabolic cost of running by injecting energy into the human-exoskeleton system. Passive support, which leverages the compliance and resilience of materials to provide a metabolic benefit, have included the Nike Vaporfly marathon shoe (4% reduction) [10]; a passive elastic hip exosuit (5% reduction) [11]; a rubber band connected between the feet (6% reduction) [12]; a passive exo-tendon hip exoskeleton (7% reduction) [13]; and a passive torsional spring hip exoskeleton (8% reduction) [14]. Powered untethered exoskeletons, however, could be especially helpful to individuals like novice or ageing runners who need larger metabolic reductions to perceive or benefit from assistance. Most recently, Witte et al. (2020) used tethered, powered ankle exoskeletons to reduce the energy cost of running by 24.7% over running in the unpowered devices and 14.6% over normal running shoes [15]. In the same study, the tethered ankle exoskeletons were used to emulate passive, spring-like ankle plantarflexion assistance, which was found to only reduce energy cost by 2.1% over running in the unpowered devices [15]. These results indicated that powered, portable ankle exoskeletons could greatly reduce the metabolic cost of running compared to passive ankle exoskeletons and have the potential to reduce energy cost more than devices that assist the hip.

The large metabolic cost reductions during running obtained in a previous study by Witte et al. (2020) were achieved by optimizing the timing and magnitude of plantarflexion assistance torque with tethered exoskeleton emulators [15]. These devices have powerful off-board motors that allow for rapid control strategy testing, thus enabling experimenters to identify energetically optimal torque assistance patterns for users in real-time through a process known as human-in-the-loop optimization [16]. Torque assistance varied as a percentage of stance time and was parameterized by magnitude of peak torque and three timing nodes: timing of torque onset (“onset time”), timing of peak torque (“peak time”), and timing of torque removal (“off time”). In the previous study by Witte et al. (2020), the optimized running assistance patterns among participants were consistently characterized by large peak assistance torque applied in late stance, but there was greater variation in onset time of assistance between participants. It is unclear how metabolic cost related to each of the optimization parameters, and how much participant customization mattered.

Translating the metabolic benefits of powered ankle exoskeletons outside of the laboratory environment introduces a tradeoff between the metabolic penalty of device mass and the amount of assistance provided to the user. If ankle exoskeletons are to become a viable product, the motors, power supply, and control system must be worn on the user. In a portable system, the mass of these components, which was unconstrained in tethered experiments, will strongly impact the user’s net metabolic cost, especially mass that is located more distally from the user’s center of mass. Furthermore, the mass of the exoskeleton end effector—here, the portion worn on the feet and shank of the user—was fixed in the tethered system but could be scaled as a function of the peak device assistance magnitude in a portable system. Kim et al. (2019) successfully demonstrated that portable, powered devices can provide a net metabolic benefit to the user by reducing the metabolic cost of running by 4% with a lightweight, soft exosuit that assists in hip extension [17]. Hip exoskeletons are an attractive approach to reducing the metabolic cost of running because the penalty associated with mass worn at the hip is low relative to mass worn at more distal locations such as the ankle. However, it is possible that portable, powered ankle exoskeletons can be carefully designed to achieve even greater reductions in the energy cost of running. To maximize the tradeoff between increasing device assistance and increasing the metabolic penalty of added device mass, it is necessary to gain a better understanding of the relationship between magnitude of ankle exoskeleton assistance and metabolic cost reduction. Characterizing this relationship could allow device designers to estimate and optimize the net metabolic benefit of candidate ankle exoskeletons.

The peak torque magnitude provided by ankle exoskeleton assistance may be strongly related to metabolic cost reductions in running. In prior studies to reduce the metabolic cost of walking and running with tethered ankle exoskeleton emulators, optimal peak assistance torques were on the higher end of the allowable range [15, 16], suggesting that peak torque has an influence on metabolic cost. Quinlivan et al. (2017) found that net metabolic rate continually decreased with increasing peak ankle exosuit assistance torque during walking, and that that this relationship fit a linear model well [18]. Characterizing the relationship between ankle exoskeleton assistance and metabolic cost in a similar manner for running, while optimizing other assistance characteristics at each peak torque level, would allow us to predict the metabolic benefit of a portable exoskeleton design.

Peak torque also directly influences the mass requirements of a portable exoskeleton through the device architecture and transmission characteristics, which in turn affects metabolic cost. The metabolic cost of running increases with device mass and its distance from the user’s center of mass [19]. Peak torque, unlike the timing of assistance, dictates the strength requirements of the exoskeleton architecture, which will strongly predict the mass of the device. A lightweight, soft exosuit that relies on shear forces to transmit assistance to the user would incur a smaller metabolic cost penalty than a heavier, framed device. However, a rigid framed device would be able to withstand higher peak torques and might more comfortably transfer those to the user with nominal shear forces. Peak torque also plays an important role in motor and transmission selection for portable device design, as motor size is strongly associated with stall torque limits [20]. Assuming similar ankle kinematics across torque magnitudes, peak torque also strongly correlates with peak mechanical power, which affects electrical energy consumption and battery size. While mechanical power is also a strong predictor of device performance, it is more difficult to systematically vary due to its dependence on human joint moment and resulting joint velocity throughout the gait cycle.

The net metabolic benefit of a portable ankle exoskeleton could be estimated and optimized using knowledge of the relationships between peak assistance torque and metabolic cost reduction, peak assistance torque and exoskeleton mass, and exoskeleton mass and metabolic cost increase. It may be that the benefits of assistance are minimal until a substantial peak assistance torque is reached. Alternatively, it may be that metabolic cost decreases linearly as peak assistance torque increases, as Quinlivan et al. (2017) found in walking [18]. It is also possible that metabolic cost does not decrease (or even increases) once a threshold of peak assistance torque is reached. For example, Kang et al. (2019) found a quadratic relationship between peak hip assistance torque and metabolic cost during slow walking, where the greatest reduction in metabolic cost was not achieved at the highest assistance magnitude [21]. Knowledge of this relationship, along with a model of device mass, would be useful to predict the net metabolic benefit of a portable ankle exoskeleton. Known relationships between user’s body weight, added mass, the location of mass placement on the user, and metabolic cost during running [19, 22] can be used to predict the mass penalty of wearing an exoskeleton. For an existing design with known peak torque capability, the metabolic benefit of assistance torque could be subtracted from the metabolic penalty of carrying the mass to predict the net metabolic cost reduction. Performing this simple analysis before building the device could save substantial time and resources by avoiding costly prototyping of inadequate devices. In the early stages of device design, researchers could also construct an optimization routine to select a motor and transmission that would maximally reduce metabolic cost.

The purpose of this study was to characterize the relationship between peak assistance torque and metabolic cost reduction during steady-state running with ankle exoskeletons. Participants underwent human-in-the-loop optimization with a bilateral ankle exoskeleton emulator at four fixed peak assistance torques. The timing of assistance torque—parameterized by onset time, peak time, and off time—was optimized at each peak torque level to minimize metabolic cost. After finding the participant’s optimized parameters, we recorded the steady-state metabolic rate of participants while running in the devices with optimized assistance (“assistance”), in the unpowered devices (“zero torque”), and in the device footwear without the exoskeleton attached (“normal shoes”). We then fit a curve to these data to estimate the relationship between peak assistance torque and metabolic cost reduction. A secondary goal of this study was to investigate how optimal timing of plantarflexion assistance varies with peak torque magnitude and participant, as well as to understand the relative effect of each timing parameter on the optimization of metabolic cost. We expect these results to inform the design tradeoffs for portable ankle exoskeletons.

## Methods

### Exoskeleton hardware and control parameterization

**Fig. 1:**
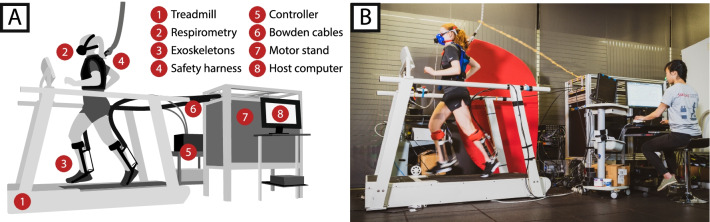
Experimental setup. **A** Exoskeleton emulator system. Exoskeleton end effectors are actuated by off-board motors via series elastic Bowden cable transmission. Respirometry data is collected by measuring the user’s oxygen consumption and carbon dioxide exhalation. **B** Participant running on the treadmill wearing tethered bilateral exoskeletons and respirometry mask

Participants wore tethered bilateral torque-controlled ankle exoskeletons with a mass of 1.1 kg each. Exoskeletons were actuated by off-board motors connected via a series elastic Bowden cable transmission (Fig. 1) [23]. Insoles with 4 force sensing resistors (Nike, Inc.) were used to detect foot strike and toe-off. Strain gauges assembled in a full Wheatstone bridge were calibrated and mounted to the end effectors to measure applied torque about the ankle joint for feedback control. An identical set of the exoskeleton footwear (Nike, Inc.) was used to evaluate the metabolic cost of running without the exoskeleton (“normal shoes”).

**Fig. 2:**
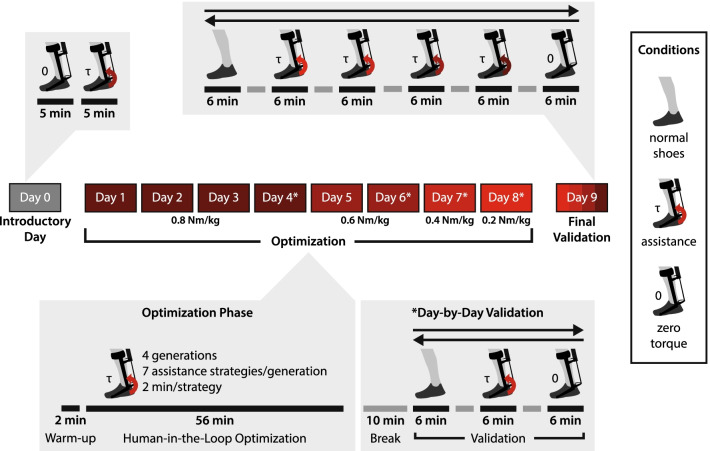
Sample experimental protocol. All participants experienced an introductory session in which they ran in the unpowered exoskeleton (“zero torque”) and with assistance. Next, participants experienced several sessions with human-in-the-loop optimization at various fixed peak assistance torque levels. (Subjects 2–3 first experienced four sessions of optimization at a peak torque level of 0.6 Nm/kg, then two sessions at 0.8 Nm/kg.) On the final day of optimization at each peak torque level (indicated by an asterisk), a series of validation trials were performed (“Day-by-Day Validation”) to compare the optimized assistance at that peak torque level to zero torque and normal shoes. During the final validation session (“Final Validation”), the optimized assistance strategy from each of the peak torque levels was tested, along with the zero torque and normal shoes conditions

The parameterization of desired torque patterns followed a similar approach to the powered assistance controller in [15], but with only three parameters that defined the timing of assistance: timing of peak torque (peak time), onset of torque (onset time), and return to zero torque (off time), all as percentages of average stance time. The peak torque magnitude, which was defined as the fourth parameter in [23], was fixed. The three timing nodes were connected by cubic spline to form an assistance torque curve.

### Participant demographics

Three recreational runners (*n* = 3; 1 F, 2 M; age: 22–41 years; body mass: 57.5–84 kg; height: 1.68–1.84 m) participated in this study (Additional file 1: Table A1). To be eligible for this extensive study design, each participant had previously run a half marathon, was running at least 20 miles per week, and could run comfortably for at least 1 h at a speed of 3.35 m/s (approximately 8 min/mile pace). These criteria ensured that the participant would be able to complete the experimental protocol in the aerobic respiration range, as the standard equation to calculate average metabolic rate is only valid for aerobic respiration [24]. All participants were consistent mid-to-rearfoot strikers at the study pace of 2.68 m/s. The study protocol was approved by the Stanford University Institutional Review Board, and all participants provided written informed consent before participating in the study. Participants were compensated 15 dollars per hour. Two additional participants were consented and participated but did not complete the study protocol due to circumstances surrounding the COVID-19 pandemic. These participants’ data were discarded.

The sample size of this study was informed by an a priori power analysis and resource tradeoffs. In a previous study, powered ankle exoskeleton assistance led to a metabolic cost reduction of 24.7 ± 6.9% relative to the unpowered condition (*n* = 11) [15]. Using this result, we found that a sample size of three participants gave a statistical power of 0.85 (two-tailed *t*-test, $\alpha$ = 0.05). Strict study inclusion criteria and lengthy protocol time (10 sessions lasting 4 h per session) favored a smaller sample size of well-trained participants. We performed two data collections per participant and per peak assistance torque level, one following optimization and one final validation session, to improve accuracy of within-participant results.

### Experimental protocol

Participants experienced 9 or 10 total experimental sessions, during which they ran on a treadmill (Woodway USA, Inc.) at a pace of 2.68 m/s (Fig. 2). Participants took at least 1 day of rest between each session. The first session was a short introductory session in which participants were introduced to the exoskeleton controller. Participants ran at least 5 min in the exoskeletons without assistance torque (“zero-torque”) followed by 5 min of generic assistance with timing similar to that from Witte et al. (2020) at a peak torque magnitude of 0.6 Nm/kg, normalized to participant body mass in kilograms [15]. Participants were encouraged to run in the devices (zero-torque or assisted) until they felt comfortable running in the exoskeletons (Additional file 2).

#### Optimization sessions

Following the introductory session, participants experienced 7 or 8 sessions of human-in-the-loop optimization to reduce metabolic rate as described in “Optimization Strategy” below. Participants fasted for 2 h before each session to reduce the thermic effect of food on metabolic rate measurements within each generation of optimization. This fasting requirement ensured that any metabolic rate measurements were taken after the initial steep increase in the thermic effect of food had passed [25]. The slow decline of the thermic effect of food on metabolic rate over the course of the study was assessed to have negligible effect on the reported metabolic results, especially because all validation trials were repeated in reverse. During each optimization, the peak assistance torque was fixed. In each experimental session, the optimization phase lasted approximately 1 h (including a 2-min warm-up with generic assistance). Following the final session of optimization at a fixed peak torque level, a series of 6-min validation trials were performed to evaluate the effects of assistance torque compared to zero-torque and normal shoes. Each experimental session lasted approximately 2.5–4 h with approximately 1–1.5 h of running.

Four peak assistance torque levels were evaluated to provide sufficient resolution across the search space within the constraints of a reasonable protocol length (up to 10 days). The highest level of peak torque that was found comfortable in pilot testing was 0.8 Nm/kg, normalized to participant body mass in kilograms. This value was slightly higher than the average optimized peak torque of 0.75 Nm/kg from a previous study [15]. The other three peak assistance torque levels were evenly spaced between the maximum value and zero torque: 0.6 Nm/kg, 0.4 Nm/kg, and 0.2 Nm/kg.

In the first 3 or 4 experimental sessions of optimization, participants experienced one of the higher assistance levels, beginning with 0.8 Nm/kg for Subject 1 and 0.6 Nm/kg for Subjects 2 and 3. The order of the first and second conditions was altered for two of the participants after the first participant experienced muscle soreness from beginning with the highest assistance level. The multi-session optimization ensured that the participants had adapted to the assistance and that the optimization parameters had fully converged. The optimization parameters were considered to have fully converged once the optimizer step size $\sigma$ (initially 10) dropped below half of the initial step size. The mean parameters from the end of the first and final session at the same assistance level were also compared to assess convergence.

After the longer optimization period at the first assistance torque level, participants then experienced 2 sessions with optimization at a different high assistance torque level (0.6 Nm/kg for Subject 1, 0.8 Nm/kg for Subjects 2 and 3). Two sessions were completed at this second assistance torque level to ensure participant adaptation translated to a different assistance torque condition, and that the optimizer had fully converged by the same step size criterion. Participants then experienced 1 session of optimization at each of the lower assistance levels (0.4 Nm/kg, then 0.2 Nm/kg). These shorter durations of optimization were a result of minimal shift in the optimized parameters and a clear downward trend in optimizer step size.

The ordering of peak torque conditions was chosen to maximize participant adaptation and facilitate optimization convergence. Poggensee and Collins (2021) found that novice users require over 100 min to learn how to maximally benefit from walking with ankle exoskeleton assistance, and that users adapt most slowly to peak torque magnitude [26]. Although all three participants in the present study had prior experience running with ankle exoskeleton assistance, additional sessions of optimization at the higher peak torque levels mitigated any effects of adaptation on the measured study outcomes. These training effects were expected to translate to lower peak torque levels. In addition, it is possible that beginning with the lowest peak torque magnitude of 0.2 Nm/kg would have resulted in poor optimization convergence. Low magnitude of assistance torque was expected to have less effect on metabolic rate, which could make it more challenging to identify the optimal timing parameters due to the higher noise-to-signal ratio and interaction effects between peak torque and timing of assistance.

During the final experimental session of optimization at a fixed peak torque level, a series of validation trials occurred after the optimization phase (“Day-by-Day Validation”), separated by a minimum break of 10 min. Each validation trial was 6 min in duration, with data collected from the last 3 min. In the first validation trial, participants stood quietly for 6 min to obtain their resting metabolic rate. The participants then ran in three separate validation conditions: assistance (the optimized assistance torque pattern at that peak torque level), zero torque, and normal shoes. These three running conditions were randomized and repeated in reverse (“bidirectional validation”) for a total of 6 running validation trials to reduce any effects of ordering on metabolic cost. Participants took at least 2 min of rest between running trials. These data were used to characterize the relationship between peak torque and metabolic cost reduction (referred to as “Day-by-Day Validation”).

In some interim sessions at the same peak torque level, validation was also performed to track participant adaptation to assistance (Additional file 1: Fig. A2). Due to some participant scheduling constraints, single-direction validation (trials were not repeated in reverse) or no validation was performed in some of these interim testing sessions. Less than 2% variation in metabolic cost reduction across experimental sessions indicated that the participant had become accustomed to running with the prescribed assistance torque level.

#### Final validation session

In the final experimental session (referred to as “Final Validation”), the effect of assistance across all 4 torque levels was compared against running with zero torque and normal shoes in a series of validation trials. No optimization occurred during this experimental session. At the beginning of the experimental session, participants stood quietly for 6 min to obtain their resting metabolic rate. Participants then ran in each of the 6 running conditions for 6 min, with data collected from the last 3 min. For each torque level, the subject-specific optimized assistance pattern was applied. The order of the running conditions was then reversed to improve measurement accuracy and reduce potential effects of ordering. The results of Final Validation were compared with the results of Day-by-Day Validation to ensure that the ordering of optimization sessions did not have an effect on participant adaptation.

### Human-in-the-loop optimization

Participants underwent human-in-the-loop optimization to determine the assistance timing parameters that maximally reduced their metabolic cost at each assistance torque level. A covariance matrix adaptation evolution strategy (CMA-ES) was used [15, 16]. Each generation of 7 candidate assistance strategies-defined by the three timing parameters discussed above-were sampled from a multivariate normal distribution about the current mean parameter set. Each candidate assistance strategy was applied to the participant for 2 min of running, during which an estimate of steady-state metabolic rate was obtained from raw respirometry data [27]. At the end of each generation, the metabolic rate results were used to update the mean parameter set and optimization state variables that defined the multivariate normal distribution. The candidate assistance strategies in the next generation were sampled from the resulting distribution.

During each optimization phase, participants experienced 4 generations of optimization for a total of 28 torque assistance strategies, equating to 56 min of running. The mean parameter set calculated from the final generation was used as the optimal assistance torque pattern at that peak torque magnitude for validation. For each participant, the optimization was initially seeded with the following set of mean timing parameters: onset time of 25% of stance, peak time of 75% of stance, and off time of 95% of stance. The optimization state variables were initialized using the same approach as [16]. The step size ($\sigma$) was initialized to 10, and the covariance matrix was initialized to the identity matrix. Peak time and off time were scaled by a factor of two to allow for finer search, as those parameters had smaller comfortable ranges than onset time during pilot testing. During experimental sessions with continued optimization at the same torque level, the optimized mean parameter set and optimization state variables were carried over from the previous experimental session. At each subsequent torque level, the optimization was seeded with the optimized mean parameter set from the previous torque level, but the optimization state variables were reset to the baseline.

During optimization, each of the three timing parameters was restricted to a range that was comfortable in pilot testing: onset time ranged from 5 to 60 percent of average stance time, peak timing ranged from 40 to 80 percent, and off time ranged from 60 to 100 percent. After a new generation of assistance strategies was sampled from the current distribution, values sampled outside of the search region were projected onto the constraint boundary. Furthermore, the onset time and off time of torque were constrained to occur at least 20 percent before and after the peak time, respectively.

### Measured outcomes

#### Metabolic rate

The reported outcomes for each condition from a single experimental session are taken as the average from the two 6-min validation trials to reduce the effects of noise. Metabolic rate, the primary outcome of this study, was measured using a respirometry system (Quark CPET, Cosmed), which was calibrated according to manufacturer instructions. Carbon dioxide and oxygen rates were measured during the last 3 min of a validation trial and substituted into a standard equation [24] to obtain average metabolic rate. The metabolic rate reported for all running validation trials was calculated by subtracting the metabolic rate of the quiet standing validation trial from the metabolic rate for the running condition. Percent reduction in metabolic rate relative to zero torque was evaluated by dividing the change in metabolic rate from zero torque by the net metabolic rate from the zero-torque condition. Percent reduction in metabolic rate relative to normal shoes was evaluated by dividing the change in metabolic rate from normal shoes by the net metabolic rate from the normal shoes condition. All metabolic rate results were normalized to participant body mass.

#### Exoskeleton mechanics

Exoskeleton mechanics were evaluated using the last 3 min of data from each validation trial. Average exoskeleton work was calculated by integrating the exoskeleton torque over ankle angle during the stance period for each stride, then averaging across all strides. Average exoskeleton power was calculated by dividing average exoskeleton work by the average stride time, as no work was performed during swing. Peak exoskeleton power was calculated as the maximum over an average gait cycle of the product of torque and ankle angular velocity. All exoskeleton mechanics were normalized to participant body mass.

#### Step frequency and duty factor

Stride and stance time for a single leg were determined from force-sensing resistors at the heel, hallux (big toe), and first metatarsophalangeal (MTP) joint. Data from the lateral MTP joint sensor was not used because it frequently stayed compressed during swing. Data were collected during the last 3 min of each assistance validation trial. At each assistance torque level, stride time and stance time data were averaged across both legs and validation sessions (Day-by-Day and Final). Step frequency (SF) was calculated from single-leg stride time and reported in steps per minute: $\text {SF} = 2 (\frac{60}{t_{\text {stride}}})$. Duty factor (DF) was calculated as the ratio of stance over stride time: $\text {DF} = \frac{t_{\text {stance}}}{t_{\text {stride}}}$.

### Statistical analyses

We pooled the data from the Day-by-Day Validation and Final Validation sessions for all participants to obtain the mean and standard deviation (SD) of the measured outcomes. To evaluate whether the level of assistance torque had an effect on the measured outcomes, we performed a mixed-effects ANOVA (fixed effect: peak torque; random effect: participant) to account for repeated measures. On measures that showed significant trends, we performed paired, two-sided t-tests comparing each assistance torque condition to the zero-torque condition from the same validation session for that participant with a Šidák-Holm stepdown correction for multiple comparisons ($\alpha$ = 0.05).

In addition, an exponential model $y = a \cdot [1 - \exp (b\cdot x)]$ was fit to the data relating peak assistance torque to percent change in metabolic rate using iterative non-linear least squares. Ninety-five percent confidence intervals (CI) were calculated for the fit parameters. Significance of the model fit was determined by an ANOVA model comparison with the constant model ($y = a$).

An asymptotic exponential model was selected over a linear model ($y = a\cdot x$) because it gives a theoretical maximum metabolic reduction that can be achieved as peak torque continues to increase. We would not expect percent change in metabolic cost to continue to decrease linearly, as the model would eventually predict an extreme at which there is no metabolic cost to run. Rather, we would expect the benefits of increasing assistance to level off as assistance replaces the contributions of the biological ankle. If the better model was indeed linear, the least squares exponential fit would be nearly linear in the region of interest and have a very large asymptote. Relative likelihood, which provides the likelihood that one model is a better fit to the data than the other, was used to compare the asymptotic exponential fit to a linear fit [28]. Relative likelihood is based on the Akaike information criterion (AIC), which estimates the relative amount of information lost by a given model using the maximum likelihood of the model. AIC also accounts for the number of parameters fit by the model, thus reducing the risk of overfitting. The relative likelihood formula for model comparison is given by $\exp \left( \frac{\text {AIC}(H_1) - \text {AIC}(H_2)}{2} \right)$. The resulting value is the likelihood that Model 2 ($H_2$) results in less information loss than Model 1 ($H_1$). The residual sum of squares for each model was also calculated to compare the asymptotic exponential model to the linear model.

The significance level for all model comparisons was $\alpha$ = 0.05. Data processing was performed using Matlab (Mathworks, Inc.) and statistical analysis was performed in R (R Core Team).

### Effect of timing parameters

To evaluate the relative importance of the three timing parameters on metabolic cost, metabolic rate estimates from human-in-the-loop optimization were recorded for each assistance strategy. Across all three participants, a total of 637 assistance strategies were tested, each of which was associated with a peak torque level, onset time, peak time, off time, and an estimate of steady-state metabolic rate. We performed a mixed-effects ANOVA ($M = t_{\text {onset}} + t_{\text {onset}}^2 + t_{\text {peak}} + t_{\text {off}}$; fixed effects: onset time ($t_{\text {onset}}$), peak time ($t_{\text {peak}}$), off time ($t_{\text {off}}$); random effects: participant, experimental session) to evaluate the effect of timing parameters on metabolic rate. Here, *M* is the steady-state metabolic rate, normalized to participant body mass (W/kg). In the optimization data, unlike the validation data, quiet standing metabolic rate was not subtracted because a measurement was not taken for every experimental session. Experimental session was treated as a random effect to capture offsets in metabolic rate between experimental days; this included metabolic rate offsets due to torque level and changes in quiet standing metabolic rate. A second-order polynomial was fit to onset time, as we would expect the cost landscape to be bowl-like. Because optimal peak time and off time were near the limit of the allowable range, the relationship between these timing parameters and metabolic rate were assumed to be linear in the region of interest. We assumed no interaction effects between timing parameters.

A single-subject pilot study (Subject 3) was conducted to further examine the effect of onset time on metabolic cost. Peak torque was fixed at 0.8 Nm/kg, and peak time and off time were fixed at the participant’s optimized values (79.7% and 100% of stance, respectively). Six minutes of quiet standing metabolic data were recorded at the beginning of the experimental session to obtain an estimate for resting metabolic rate. The participant then ran in a series of 6-min validation trials under six conditions: four conditions to sweep torque onset time (5%, 15%, 25%, and 35% of stance), zero-torque, and normal shoes. The order of these running trials was randomized. The running trials were repeated in reverse order for a total of 12 trials. The measured outcome of this pilot study was the percent reduction in metabolic rate over the zero-torque condition. A second-order polynomial least squares model was fit to the single-subject pilot study data relating onset time to percent reduction in metabolic rate. The adjusted R-squared value is reported in addition to the model significance.

## Results

### Exoskeleton mechanics

**Fig. 3:**
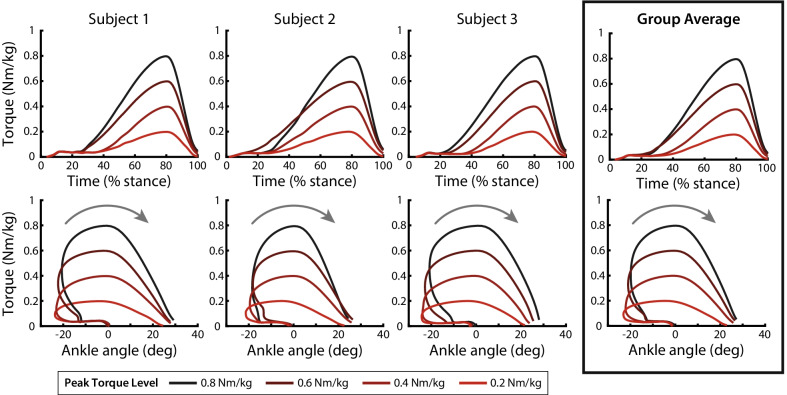
Optimized assistance patterns. *Top row:* Individual and group average of the measured torque assistance profiles as a percentage of stance. Peak torque levels are represented as different shaded lines, with 0.8 Nm/kg as the darkest. Similar timing strategies were preferred across subjects. *Bottom row:* Torque versus ankle angle curves show a large amount of net work (represented by the area below the curves) that increases with peak torque level

Human-in-the-loop optimization converged rapidly to late peak timing and off timing of assistance torque for all participants and across all torque levels (Fig. 3). At assistance torque levels with multiple sessions of optimization, mean parameters were similar after the first and final sessions of optimization. Optimized peak time shifted by an average of 0.7% of stance (1.5% maximum) and optimized off time shifted by an average of 0.4% of stance (0.8% maximum). Onset time shifted by an average of 3.3% of stance (9% maximum), although further analysis suggests that onset time had little effect on metabolic cost. These results indicate that convergence was achieved within a single session of optimization. The step size convergence criterion (half of the initial step size) was met for all multi-session optimizations, and the step size showed a strong decreasing trend for single-session optimization.

**Fig. 4:**
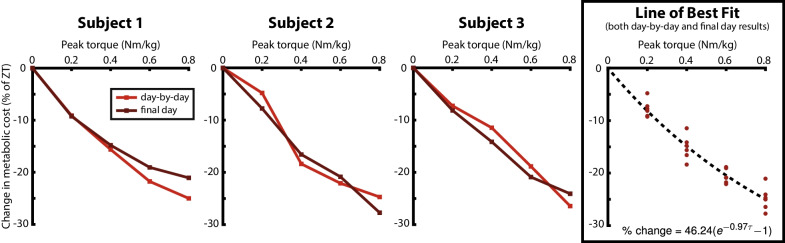
Metabolic results. Metabolic cost decreased with increasing peak torque assistance for all subjects. In the Subject-Specific plots, the final day validation results are shown in a darker-colored line than the day-by-day results following optimization. In the Line of Best Fit plot, the relationship between peak torque and change in metabolic cost is well approximated by an exponential fit in the tested range of peak torque values. The decaying exponential model fit the data better than a constant model (ANOVA; p = 6e–14)

Optimized timing of peak torque converged to a value near the latest allowable (80% of stance) at 79.3 ± 0.86% (mean ± SD) of stance. Optimized timing of torque removal converged to a value near the latest allowable (toe-off, or 100% of stance) at 99.8 ± 0.26% of stance. The timing of torque onset saw more variation, occurring at 22.5 ± 7.75% of stance. There was greater variation in torque onset time across torque levels than between participants, and later torque onset time was associated with lower peak torque magnitudes (Table 1).

**Table 1:** Optimal parameters

Peak torque magnitude	Onset time (% stance)	Peak time (% stance)	Off time (% stance)
0.2 Nm/kg	28.92 ± 5.56	78.25 ± 1.07	99.64 ± 0.32
0.4 Nm/kg	27.51 ± 6.68	79.51 ± 0.52	99.71 ± 0.38
0.6 Nm/kg	15.23 ± 6.59	79.89 ± 0.15	99.98 ± 0.03
0.8 Nm/kg	18.15 ± 1.97	79.75 ± 0.25	99.93 ± 0.10
All conditions	22.45 ± 7.75	79.35 ± 0.86	99.82 ± 0.26

Similar peak and off time of torque assistance were preferred across peak torque magnitudes. Onset time had the most variation across torque magnitudes and between subjects

Average exoskeleton mechanical power (P) increased with peak torque and was well-approximated by a linear relationship (linear mixed-effects model; $\text {P}_{\text {avg}} = 0.754\tau _{\text {peak}} - 0.007$; $\text {P}_{\text {avg}}$ (W/kg), $\tau _{\text {peak}}$ (Nm/kg); marginal $R^{2}$ = 0.98, p < 2e–16). Peak exoskeleton mechanical power also increased linearly with peak torque (linear mixed-effects model, $\text {P}_{\text {peak}} = 10.04\tau _{\text {peak}} - 0.24$; $\text {P}_{\text {peak}}$ (W/kg), $\tau _{\text {peak}}$ (Nm/kg); marginal $R^{2}$ = 0.97, p < 2e–16) (Table 2).

**Table 2:** Exoskeleton mechanics

Peak torque magnitude	Average work (J/kg)	Average mechanical power (W/kg)	Peak mechanical power (W/kg)
0.2 Nm/kg	0.10 ± 0.01	0.14 ± 0.01	1.75 ± 0.14
0.4 Nm/kg	0.23 ± 0.01	0.30 ± 0.01	3.79 ± 0.31
0.6 Nm/kg	0.35 ± 0.02	0.45 ± 0.02	5.82 ± 0.35
0.8 Nm/kg	0.46 ± 0.04	0.59 ± 0.03	7.77 ± 0.47

Average work, average mechanical power, and peak mechanical power increased with increasing peak torque magnitude

### Metabolic rate

Net metabolic rate decreased as the peak torque magnitude of exoskeleton assistance increased (mixed-effects ANOVA; p = 2e-12; Fig. 4; Table 3). In the Final Validation session, metabolic rate results were similar to the results from Day-by-Day Validation sessions. By the end of the first experimental session, all participants had adapted quickly to assistance, showing less than 2% variation in metabolic rate reduction between the first and final session of optimization at the same torque level (Additional file 1: Fig. A2). Participants did not exceed a steady-state respiratory exchange ratio of 1.0, which indicates that they completed the entire study in the aerobic range.

**Table 3:** Net Metabolic Rate

Assistance condition	Net metabolic rate (W/kg)	Metabolic rate reduction (% of ZT)
Normal shoes	8.76 ± 0.42	12.2 ± 2.7%
Zero torque	9.99 ± 0.66	–
0.2 Nm/kg	9.20 ± 0.59	7.7 ± 1.6%
0.4 Nm/kg	8.50 ± 0.57	15.2 ± 2.3%
0.6 Nm/kg	7.85 ± 0.56	20.6 ± 1.4%
0.8 Nm/kg	7.56 ± 0.70	24.8 ± 2.3%

Net metabolic rate (quiet standing rate subtracted) decreased with increased peak torque magnitude. The metabolic rate reduction is reported as a percentage of zero torque from the same validation

The net metabolic rate with optimized assistance torque at the highest peak torque magnitude of 0.8 Nm/kg was 24.8 ± 2.3% (mean ± SD) lower than running with the exoskeletons in zero torque mode (*t*-test, p = 4e–6). At subsequent peak torque magnitudes of 0.6 Nm/kg, 0.4 Nm/kg, and 0.2 Nm/kg, the net metabolic rates with assistance were 20.6 ± 1.4% (*t*-test, p = 1e–6), 15.2 ± 2.3% (*t*-test, p = 4e–5), and 7.7 ± 1.6% (*t*-test, p = 8e–5) lower than running in zero torque mode, respectively. The net metabolic rate of running with the highest assistance torque magnitude (0.8 Nm/kg) was 14.1 ± 3.8% lower than running in normal shoes (*t*-test, p = 3e–4).

The relationship between percent reduction in net metabolic rate and peak assistance torque magnitude is well-approximated by a decaying exponential curve of the form: $\text {\% reduction} = a \cdot [1 - \exp (b \cdot \tau _{\text {peak}})]$, $a =$-46.24 (p = 1e–4, CI = [25.64, 66.84]), $b =$-0.97 (p = 3e–3, CI = [− 1.57, − 0.38]) (Fig. 4). The exponential model fit the data better than a constant model (ANOVA, p = 6e–14). Furthermore, the relative likelihood of the decaying exponential model with respect to a linear model ($\text {\% reduction} = c \cdot \tau _{\text {peak}}$, where $c = - 33.19$) was 108.6. Thus, the decaying exponential model is 108.6 times as probable as the linear model to minimize the loss of information [28]. The residual sum of squares of the decaying exponential fit was 77.33, which was lower than the residual sum of squares of the linear fit of 124.2.

### Step frequency and duty factor

Step frequency (SF) decreased as peak assistance torque increased (linear mixed-effects model; $\text {SF} = 162 - 10.6\tau _{\text {peak}}$; SF (steps/min), $\tau _{\text {peak}}$ (Nm/kg); conditional ${R^{2}}$ = 0.98, p = 2e–5), resulting in a gait with more “push” characteristics as described by Oeveren et al. (2021) [29]. To obtain an estimate for step frequency for a new participant, step frequency in the provided equation should be scaled to the new participant’s leg length by multiplying by a factor of $0.914/L_0^{1/2}$, where $L_0$ is the new participant’s leg length from the greater trochanter to lateral malleolus in meters [30]. Duty factor also decreased with peak assistance torque (linear mixed-effects model; $\text {DF} = 0.38 - 0.036\tau _{\text {peak}}$; p = 0.001, conditional ${R^{2}}$ = 0.97), which is indicative of a running style with more “bounce” characteristics [29].

### Effect of timing parameters

A mixed-effects model (fixed effects: onset time, peak time, off time; random effects: participant, experimental session) was fit to data from human-in-the-loop optimization to evaluate the effect of timing parameters on the steady-state metabolic rate of running ($M = 17.04-0.047 t_{\text {onset}} + 0.31 t_{\text {onset}}^2-0.028 t_{\text {peak}}-0.052 t_{\text {off}}$). We found that peak time (ANOVA, p = 2e-6) and off time (ANOVA, p = 2e–7) had strong effects on metabolic rate, but onset time did not (ANOVA, p = 0.64).

In the single-subject pilot study to sweep onset time across 5, 15, 25, and 35% stance, the net metabolic rate was reduced by 23.9 to 26.2% compared to running in the zero-torque condition (Additional file 1: Fig. A1). The participant was unable to accurately rank the ordering of onset time trials but described the 35% onset as “a little less comfortable” than earlier onset times. Onset time did not have an effect on percent reduction in metabolic cost, as a quadratic model did not fit the data better than a constant value (second-order polynomial least-squares fit; $\text {\% reduction} = -24.95-1.16 t_{\text {onset}} + 0.90 t_{\text {onset}}^2$; ANOVA, p = 0.48; adjusted ${R^{2}}$ = 0.31).

## Discussion

Increasing peak exoskeleton assistance torque up to 0.8 Nm/kg led to a slightly nonlinear decrease in percent change in the metabolic cost of running for all participants, with diminishing returns at higher torques. The effectiveness of these large torques is consistent with the results of a previous human-in-the-loop optimization study in which peak torque could vary as an optimization parameter, which found that high peak torque magnitudes were preferred by the optimizer for most participants [15]. In the present study, the averaged data across participants show that the effects of assistance began to level out slightly at the highest tested torque level, with a relationship that is well-approximated by a decaying exponential (Fig. 4). The asymptote of this curve gives a theoretical maximum metabolic reduction (46%, CI: 26–67%) that can be achieved with ankle exoskeleton assistance, if the peak torque were to exceed the maximum level applied in this study. However, peak torque was limited to 0.8 Nm/kg due to user stability preferences, and there may be little value in designing devices that exceed this limit if users are not able to adapt to higher torque magnitudes. These results suggest that the net metabolic benefit of running with an untethered device will likely be optimized at a value of peak torque near the higher end of the tested range (0.6–0.8 Nm/kg). In the absence of a user-preferred peak torque limit, continuing to increase peak assistance torque would eventually result in a decrease in net improvement, as device mass is expected to continually increase with peak torque, whereas the benefits of assistance level off.

Additional training may allow participants to tolerate higher torques and achieve even larger reductions in energy cost, up to some user-preferred limit. Previously, Witte et al. (2020) found that some participants did not optimize to the maximum allowable peak torque magnitude [15]. This suggests that, for users at some stages of training, large assistance torques can increase metabolic cost, perhaps by requiring additional stabilizing muscle activity through, e.g., co-contraction [31]. More recently, Poggensee and Collins (2021) found that naïve exoskeleton users required much longer training periods to become expert, more than 100 min of exoskeleton exposure [26]. In the present study, experiencing optimization sessions fixed at a peak torque magnitude of 0.8 Nm/kg may have allowed for more complete motor learning, enabling participants to discover less metabolically costly solutions that afforded similar levels of stability [32]. While our results predict that further increasing peak assistance torque would lead to greater reductions in metabolic cost, the peak torque magnitude was limited to 0.8 Nm/kg because participants expressed that they felt unstable at a high peak torque magnitude. One participant was involved in pilot testing at 1.0 Nm/kg and had difficulty running on the treadmill without mis-stepping. They experienced muscle soreness that they attributed to “bracing to stay in the same place on the treadmill”. Another participant stated that running with peak assistance torque of 0.8 Nm/kg made it “harder to control the device” and felt that they “had to be very consistent about landing and launching.” This feedback suggests that the cost function that the exoskeleton user is trying to optimize contains additional terms besides metabolic cost such as perceived instability and level of discomfort. It is possible that participants would feel comfortable with-and might even prefer-higher levels of assistance at faster speeds or in less-constrained environments such as overground running. The limit of peak torque magnitude might also vary on an individual basis, and scaling peak torque with the user’s body mass might not capture other participant characteristics. Future research could investigate how user-preferred torque limits change with speed, environment, and duration of optimization.

Late peak time and off time of torque were consistently preferred by all participants across all torque levels. During the first session of human-in-the-loop optimization, peak timing and off timing of torque quickly shifted within a single generation from the initial seed (75% and 95% of stance, respectively) toward the latest allowable (80% and 100% of stance). This preference for the latest allowable torque application was maintained across optimization sessions for all peak torque magnitudes. The final optimized peak time and off time parameters were consistent across participants and peak torque magnitudes within a standard deviation of less than 1% of stance (Table 1). These results support the observation that biomimetic assistance with a peak around 50% of stance is not metabolically optimal [15]. A similar trend is observed in walking; greater metabolic benefit is achieved by providing peak torque assistance later in stance than peak biological ankle moment [16, 33]. Rather, it is more effective to assist in late stance when force-generating capacity of the plantarflexor muscles is limited due to high shortening velocity and sub-optimal muscle fascicle length [34, 35].

Onset time within the tested range appeared to have little effect on metabolic cost, especially compared to the timing of peak torque and torque offset. Onset time varied more greatly across torque levels than between participants, with later torque onset favored at lower torque levels. While this variation might suggest a need for customization according to torque level and participant, onset time was also slow to converge to the optimized values compared to peak time and off time. This phenomenon shows that there might not be a clear optimal onset time; small differences in metabolic rate between onset time conditions might have been dominated by noise in the metabolic rate estimates, causing the optimizer to converge more slowly. Furthermore, the data from various assistance strategies tested during human-in-the-loop optimization indicates strong effects of peak time (p = 2e–6) and off time (p = 2e–7) on metabolic cost, but no clear effect of onset time (p = 0.64). Similarly, the single-subject pilot study to explore the metabolic effects of onset time found little variation in metabolic cost, and there was no clear optimal value for onset time. This supports the conclusion that onset time across the search range used in this study has little effect on metabolic cost compared with peak time and off time of assistance torque. The optimized onset timing from human-in-the-loop optimization was likely part of a range of values that would have resulted in a similar metabolic cost. Future research could be conducted to generalize this claim across more participants and assistance torque magnitudes for all three timing parameters. It is likely that there are ranges for peak time and off time that are similarly effective in reducing metabolic cost, although these ranges are expected to be much smaller (e.g., 1% of stance) than the range of onset time. In a future study, each timing parameter could be swept individually to determine the effects on metabolic rate, or a grid search could be conducted to also assess interaction effects between timing parameters (and with peak torque as well), which were not considered in this analysis.

The consistency in timing and metabolic results across participants suggests that users could obtain near-optimal metabolic benefits from an exoskeleton that provides generic assistance timing. Customization of assistance to individual users is less suited to the development of a portable exoskeleton that can be made commercially available, especially if it might have a very weak effect on metabolic cost. The results from the present study indicate that late timing of peak torque (80% of stance) and torque offset (100% of stance) would be well suited for a range of participants. The weak effects of torque onset time on metabolic cost from analysis of the human-in-the-loop optimization data indicates that a generic, comfortable onset time (e.g., 20% of stance) could be similarly beneficial across users. Longer training periods, especially for novice users, may be required for users to maximize metabolic benefit from a generic assistance profile.

The results of this study suggest that a portable ankle exoskeleton capable of high peak torque in the range of 0.6–0.8 Nm/kg might deliver the maximum net metabolic benefit relative to running in normal shoes, and that low levels of ankle assistance might not provide a worthwhile benefit. At the lowest peak torque level of 0.2 Nm/kg, the 7.7% metabolic benefit of assistance did not exceed the 8% net benefit achieved by the most effective portable, passive hip exoskeleton to date [14]. Thus, it would not make sense to design portable ankle exoskeletons for this low assistance torque level, as the device would need to be nearly massless to have a comparable effect to existing portable technologies. However, running with the highest peak assistance torque level of 0.8 Nm/kg in tethered exoskeletons (1.1 kg each), which were not mass-optimized, led to a 14.1% decrease in metabolic cost relative to running in normal shoes. A portable exoskeleton design would need to incorporate the added mass of an onboard actuation system, thus increasing metabolic cost. However, it is likely that the frame mass could be significantly reduced to avoid a large increase in metabolic penalty, because the tethered devices used in this study were designed to withstand higher torque magnitudes. It is possible that a portable ankle exoskeleton could be constructed with a similar mass on the lower leg and an additional 2–3 kilograms at the waist for a power supply and control unit, which would incur an additional 2–3% metabolic penalty [19]. These simple considerations suggest that a metabolic cost reduction of more than 10% could be achieved by a powerful portable exoskeleton.

Quantifying the relationship between peak assistance torque and metabolic cost will allow designers of ankle exoskeletons to better estimate the net metabolic benefit of a portable device. Instead of costly prototyping and human subject experiments to assess exoskeleton performance, researchers could simply estimate the peak torque capability and mass distribution of an existing device design. If the expected net metabolic benefit did not match the desired result, the design could be modified or abandoned before investing time and resources. This framework can also be used to optimize the design of portable running exoskeletons. A designer could select a baseline exoskeleton architecture and transmission, then tune device characteristics to achieve a desired assistance torque profile. Design parameters such as the device lever arm or gearbox ratio could be optimized to minimize the metabolic effect of device mass. Simple motor analyses could be performed to select the minimum mass motor that achieves the desired assistance torque profile. By repeating this process for a range of peak assistance torques and using the known metabolic benefits of assistance from the present study, the designer could construct a curve that relates net metabolic benefit of the device to peak assistance torque. The exoskeleton design resulting in the greatest net metabolic benefit across assistance torque levels could then be fabricated and tested.

This study has some important limitations that provide directions for future work. The small number of participants in this study limits the degree to which these results can be generalized to all runners. Furthermore, the participants were experienced runners with similar fitness levels and may not be representative of other groups like novice runners or individuals with different body compositions. Future studies could test whether these results hold among a more representative sample of runners.

While this study characterizes the relationship between peak assistance torque and metabolic cost, other aspects of exoskeleton assistance have implications for both metabolic benefit and device design. In this study, the shape of the assistance curve was constrained by cubic splines fit to three parameterization nodes. We would expect that altering the basis function of the assistance curve would change the metabolic benefit of assistance, while also strongly influencing the best transmission characteristics for a portable exoskeleton. Another aspect of assistance that was not explored was altering the control architecture, such as varying torque with ankle angle, which would emulate the incorporation of passive elements like springs or clutches into a portable device. While emulating pure spring-like assistance for running did not lead to a large reduction in metabolic cost in [15], and in the present study the exoskeleton work loops were nearly monotonic (Fig. 3), future work might explore how passive device elements might work in tandem with powered assistance to maximize metabolic benefit and reduce device mass and power requirements.

Understanding optimal ankle exoskeleton assistance for running can help designers of exoskeletons to develop lightweight, powered exoskeletons that can greatly reduce metabolic cost. Portable exoskeletons that make running easier could greatly improve accessibility to running, encouraging physical activity and enjoyment of the sport. In the future, running exoskeletons might even help populations with muscle weakness or mobility limitations to keep up with able-bodied friends. We expect this work will inform the design of untethered devices and experiments in a community setting, where these potential benefits can be studied.

## Conclusions

We found that the metabolic cost of running with tethered ankle exoskeleton assistance decreased with increasing magnitude of peak assistance torque up to a user-preferred limit of 0.8 Nm/kg. The metabolic benefits of increasing assistance torque, quantified by percent reduction in metabolic rate, began to diminish at higher peak torque levels, which was well-approximated by an asymptotic exponential curve. When designing portable exoskeletons for running, the maximum net metabolic benefit might be achieved by devices that can provide large peak assistance torques in the range of 0.6–0.8 Nm/kg. Similar late timing of assistance torque was preferred across participants and assistance torque magnitudes, which suggests that an exoskeleton product with generic timing of assistance could provide near-optimal metabolic benefit across users.

## Supplementary information

**Additional file 1.** This file provides additional data not shown in figures and tables in the main text.** Figure A1** shows the metabolic rate results of a onset time sweep from a single participant. Figure A2 shows the metabolic rate results from validation trials from all experimental sessions.** Table A1** provides participant demographic information, including sex, mass, age, and height.** Table A2** provides raw metabolic rate results (in W/kg) for all participants in each condition, both from day-by-day and final validation.

**Additional file 2.** In this video, a participant runs with the highest magnitude of peak assistance torque (0.8 Nm/kg). The video is slowed down by a factor of 8 to demonstrate the exoskeleton assistance.

**Additional file 3.** This file provides a subset of the study data for each participant, including raw metabolic rate from validation; net metabolic rate from validation; exoskeleton work and mechanical power from validation; optimal parameters for each peak torque condition from human-in-the-loop optimization; mean parameter set and step size (σ) from each generation of human-in-the-loop optimization; and ordering of validation conditions. Additional data and detailed descriptions of these data are available from the corresponding author on reasonable request.

## Data Availability

A subset of the study data are included in Additional file 3. Additional data for this study are available from the corresponding author on reasonable request.

## Abbreviations

AIC: Akaike information criterion
ANOVA: Analysis of variance
CI: Confidence interval
CMA-ES: Covariance matrix adaptation evolution strategy
COVID-19: Coronavirus disease of 2019
CPET: Cardiopulmonary exercise test
DF: Duty factor
HILO: Human-in-the-loop optimization
MTP: Metatarsophalangeal
SD: Standard deviation
SF: Step frequency
