# Supplementary material for: Characterizing the relationship between peak assistance torque and metabolic cost reduction during running with ankle exoskeletons
Source: J Neuroeng Rehabil. 2022 May 12;19:46. doi: 10.1186/s12984-022-01023-5 (PMC9096774; doi:10.1186/s12984-022-01023-5)
Supplement: Supplementary file 1 — Additional file 1. This file provides additional data not shown in figures and tables in the main text. Figure A1 shows the metabolic rate results of a onset time sweep from a single participant. Figure A2 shows the metabolic rate results from validation trials from all experimental sessions. Table A1 provides participant demographic information, including sex, mass, age, and height. Table A2 provides raw metabolic rate results (in W/kg) for all participants in each condition, both from day-by-day and final validation. [file 12984_2022_1023_MOESM1_ESM.docx]

**Additional file 1 ­– Supplementary Figures & Tables**


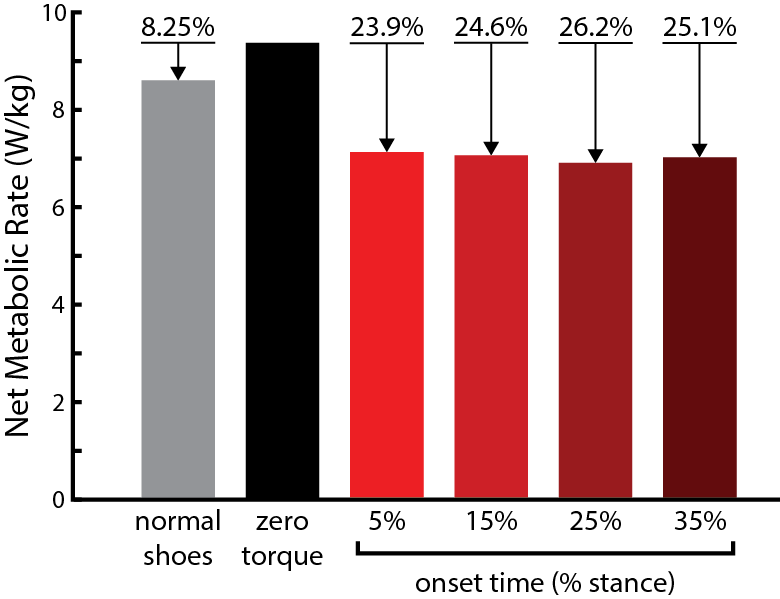


**Figure A1. Onset Time Sweep.** A single-subject (S3) pilot study was conducted to assess the effects of onset time on net metabolic rate. Four onset time conditions were tested with the same peak torque magnitude (0.8 Nm/kg, 67.2 Nm), peak timing (79.74% of stance), and off timing (99.99% of stance). These fixed timing parameters matched the optimized parameters for the 0.8 Nm/kg peak torque condition. Net metabolic rate was very similar across onset time conditions. The greatest metabolic cost reduction was achieved with torque onset at 25% of stance rather than the condition closest to the optimized parameter from human-in-the-loop optimization (15.88% of stance). These results suggest that the variation in onset time had little to no effect on metabolic cost for this subject, although further work is required to assess this claim across subjects.

**
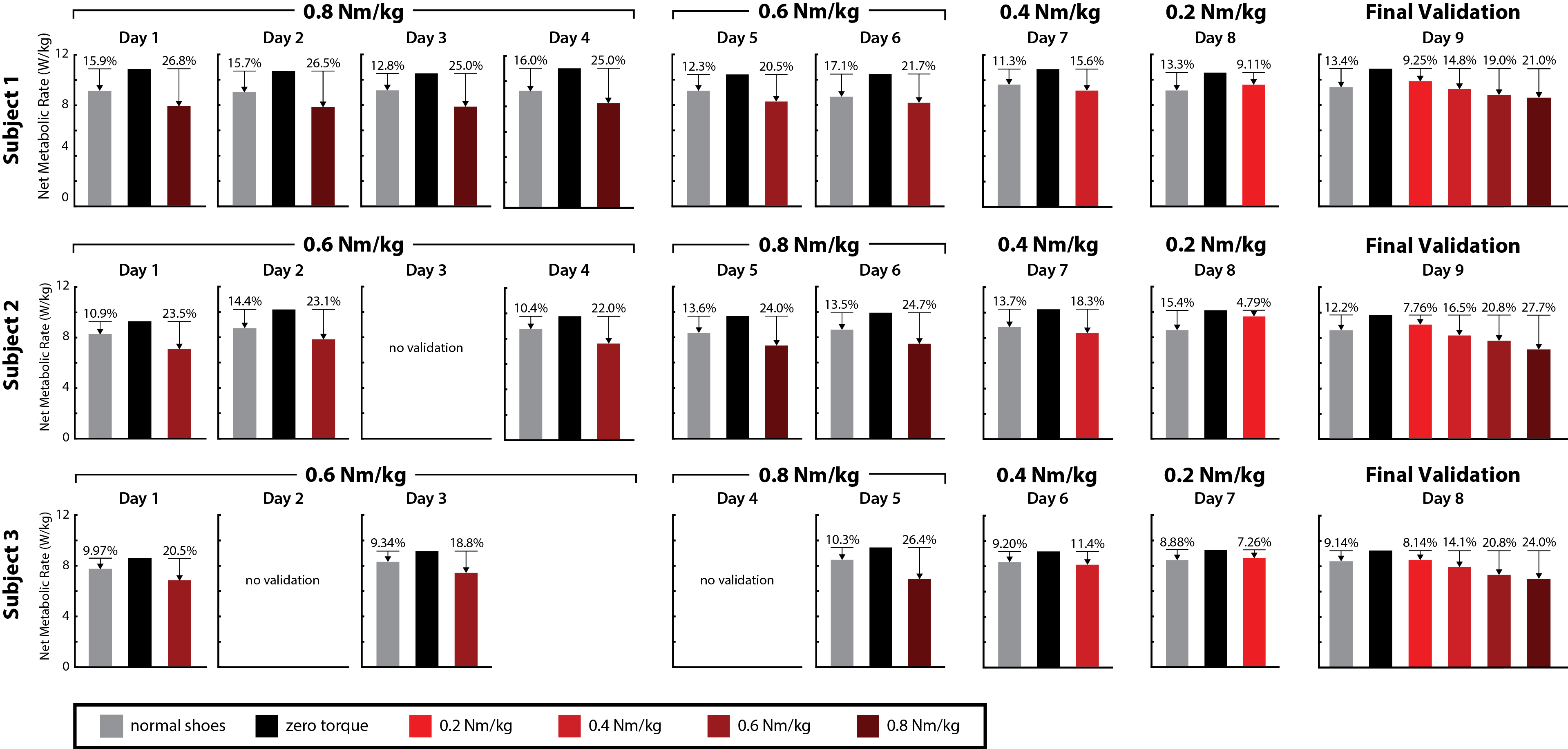
**

**Figure A2. Day-by-Day Metabolic Rate Results.** Net metabolic rate results from validation all experimental days are shown. Net metabolic rate is normalized to body mass (W/kg), and percentages indicate reductions from running in the unpowered device (“zero torque”). Following a rest break after human-in-the-loop optimization, three validation conditions (normal shoes, zero torque, and optimized torque) were tested for 6-minute running trials in a random order. The conditions were repeated in reverse order for a total of 6 trials (“double validation”). On some intermediate days of continued optimization at the same peak assistance torque level, single-direction or no validation was performed due to time constraints. During “Final Validation”, participants did not undergo human-in-the-loop optimization; they experienced 6-minute running trials of all four optimized torque conditions (0.2 Nm/kg, 0.4 Nm/kg, 0.6 Nm/kg, and 0.8 Nm/kg) in addition to normal shoes and zero torque for a total of 6 validation conditions. These conditions were repeated in reverse order.


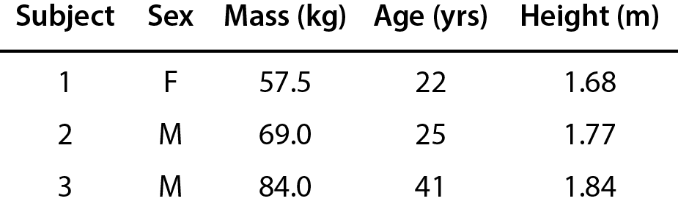


**Table A1. Subject Characteristics.** Relevant characteristics for study participants.

**
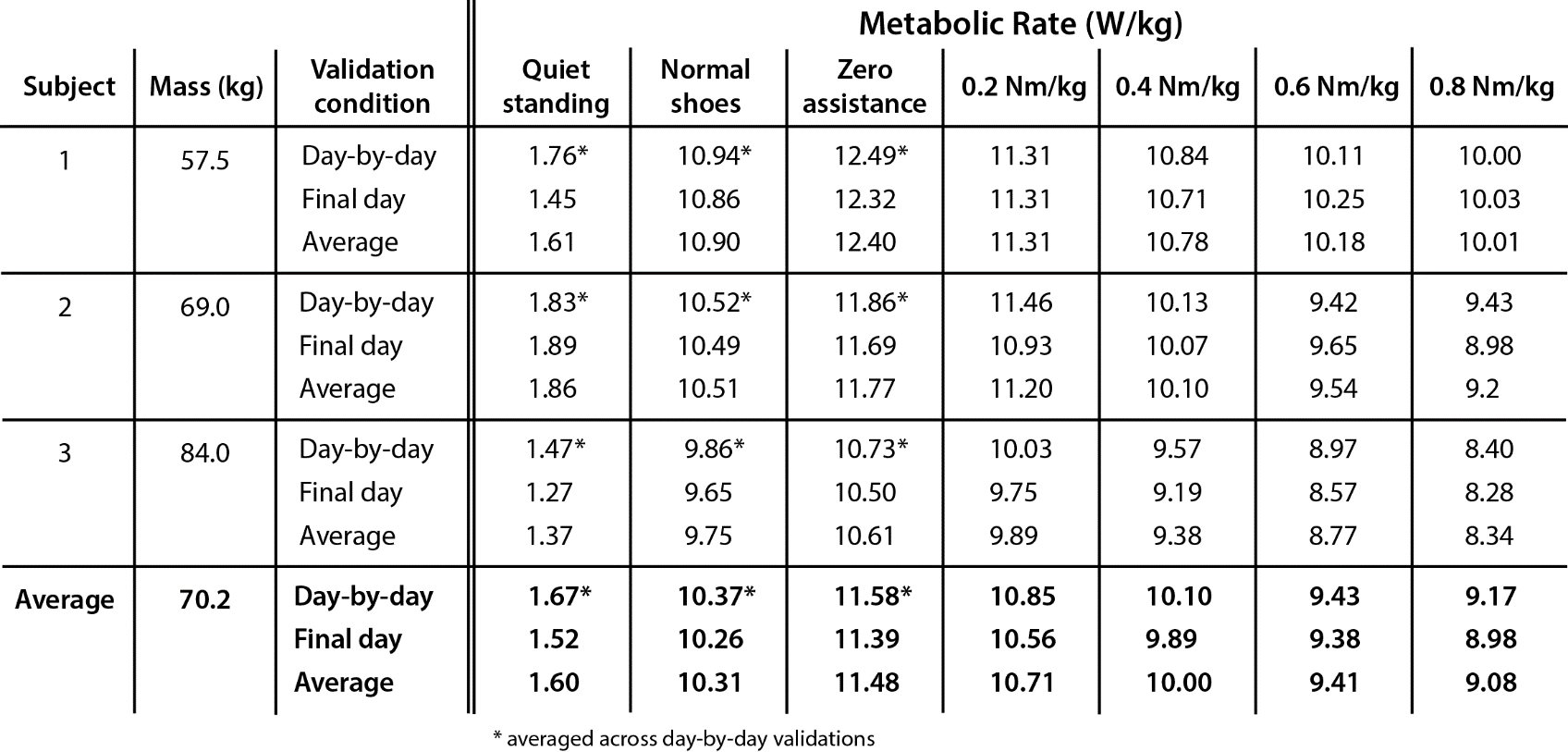
**

**Table A2. Raw Metabolic Rate.** Metabolic rate without quiet standing subtracted is reported for all participants across conditions. Values marked with an asterisk are taken as the average across all 4 day-by-day validations.
